# Can home care for homebound patients with chronic heart failure reduce hospitalizations and costs?

**DOI:** 10.1371/journal.pone.0182148

**Published:** 2017-07-28

**Authors:** Boris Punchik, Roman Komarov, Dmitry Gavrikov, Anna Semenov, Tamar Freud, Ella Kagan, Yury Goldberg, Yan Press

**Affiliations:** 1 Home Care Unit, Clalit Health Services, Yasski Clinic, Beer-Sheva, Israel; 2 Unit for Community Geriatrics, Division of Health in the Community, Ben-Gurion University of the Negev, Beer-Sheva, Israel; 3 Clalit Health Services, Southern District, Kenion Ha Negev Towers, Beer-Sheva, Israel; 4 Department of Family Medicine, Siaal Family Medicine and Primary Care Research Center, Faculty of Health Sciences, Ben-Gurion University of the Negev, Beer-Sheva, Israel; Kurume University School of Medicine, JAPAN

## Abstract

**Background:**

Congestive heart failure (CHF), a common problem in adults, is associated with multiple hospitalizations, high mortality rates and high costs.

**Purpose:**

To evaluate whether home care for homebound patients with CHF reduces healthcare service utilization and overall costs.

**Methods:**

A retrospective study of healthcare utilization among homebound patients who received home care for CHF from 2012–1015. The outcome measures were number of hospital admissions per month, total number of hospitalization days and days for CHF only, emergency room visits, and overall costs. A comparison was conducted between the 6-month period prior to entry into home care and the time in home care.

**Results:**

Over the study period 196 patients were treated by home care for CHF with a mean age of 79.4±9.5 years. 113 (57.7%) were women. Compared to the six months prior to home care, there were statistically significant decreases in hospitalizations (46.3%), in the number of total in-hospital days (28.7%), in the number of in-hospital days for CHF (66.7%), in emergency room visits (47%), and in overall costs (23.9%).

**Conclusion:**

Home care for homebound adults with CHF can reduce healthcare utilization and healthcare costs.

## Introduction

Congestive heart failure (CHF) is a clinical condition that stems from a structural or functional problem in the heart and impairs the capacity of the ventricles to fill or eject blood[1]. This condition is very common in the general population[1–8]. According to various estimates about 5.1–5.8 million people in the United States[1, 2, 7] and about 26 million people around the world [6] suffer from CHF. In some countries patients with CHF comprise 1–3% of the general population[3, 4, 6, 7]. The prevalence of CHF increases with age and reaches 8.4% among individuals 75 years of age and above and 17.4% in 85 year olds[3–5, 9, 10], so more than 80% of CHF patients are 65 years of age or older[6]. CHF impairs the quality of life of patients and their families, causes anxiety and depression[6], social isolation and a sense of loss of control[11]. CHF is the most common cause of hospitalization in the 65+ age group in the US and Europe[2] with a rate of repeat hospitalizations within one month of discharge from the hospital of 18–27%[1, 3–6, 12–16] and 50% within the first half year after discharge[5, 6]. CHF has high mortality rates[1, 3, 17] at 9–11% over the first month following diagnosis[5, 12], 20–37% in the first year[5, 12], and 45–60% in the first five years[18]. The high morbidity and mortality rates engender high costs. About 1–2% of the national healthcare costs in the United States are for CHF with the cost of hospitalization representing about 80% of this cost[3, 5].

Because of the high morbidity and mortality rates, the high level of suffering for patients and families, and high healthcare costs, significant efforts are being invested in the development of intervention programs. The aims of these programs are to improve the quality of care and the quality of life of patients, reduce mortality rates[19, 20], reduce the number of hospitalizations[10, 15, 19–22], and decrease healthcare costs[19, 21]. Several systematic reviews and meta-analyses on the prevention and management of CHF have been published in recent years. The findings of these studies have led to recommendations of professional groups[1, 6] or the prevention and treatment of CHF by multidisciplinary teams.

The aim of the present study was to evaluate the effect of home care for homebound CHF patients on healthcare utilization and overall cost.

## Methods

### Setting

The home care unit of the Clalit Healthcare Services in southern Israel has been active since 2012. One of its main aims is the treatment of homebound CHF patients in the framework of home support care. The unit’s multidisciplinary team is comprised of cardiology consultants, internists, family physicians (some of who are also certified in geriatric medicine), nurses, nutritionists, a social worker, and physical and occupational therapists.

Patients, who were homebound due to severe CHF were referred to the unit by their primary physician. The diagnosis of CHF, in most cases, was made by the primary care physician or by a physician from the hospital during a prior hospitalization. The diagnosis was verified by the unit team according to the criteria of New York Heart Association functional classification[23] (**Class I:** No limitation of physical activity. Ordinary physical activity does not cause undue fatigue, palpitation, dyspnea; **Class II:** Slight limitation of physical activity. Comfortable at rest. Ordinary physical activity results in fatigue, palpitation, dyspnea; **Class III:** Marked limitation of physical activity. Comfortable at rest. Less than ordinary activity causes fatigue, palpitation, or dyspnea; **Class IV:** Unable to carry on any physical activity without discomfort. Symptoms of heart failure at rest. If any physical activity is undertaken, discomfort increases). After the referral note and the patient’s computerized medical record were received at the home care unit, one of the four geriatricians who work in the home care unit made a preliminary house call where they conducted a comprehensive geriatric assessment that included physical condition, emotional state, nutritional state, functional status, mobility and safety in the home. CHF patients who met the criteria for treatment in the framework of home care for CHF (NYHA class 3–4, did not live alone, consented to home care) were admitted to the program. The treating team included a physician, a nurse and, if appropriate, additional personnel such as a dietician, a social worker, a physical therapist, an occupational therapist, and a cardiology specialist.

The principal of treatment in the unit was a comprehensive biopsychosocial approach to the patient’s problems. The unit staff was responsible for the patient’s care and was available to the patient and their principal caregiver at all times. The staff encouraged the patient to fulfill all early medical directives.

The uniform medical record was maintained on unit laptop computers that could be accessed by all members of the medical staff. The staff members maintained ongoing communication about the patient including combined house calls, telephone conversations and regular staff meetings that were held at least once each month. The treating team developed a comprehensive treatment plan for the patient that included oral and intravenous drug therapy (diuretics, iron), appropriate nutrition, adjusted living conditions, a personal physical therapy plan, emotional support, realization of social rights, etc.

The patients were requested to keep a daily record of blood pressure measurements and weight. The frequency of house calls was based on the patient’s clinical needs. In the event that the patient was hospitalized the medical staff updated its comprehensive assessment following discharge from the hospital with the aim of adapting treatment to changes in the patient’s condition.

Treatment in the unit was not limited in time. It was based only on the patient’s clinical needs and their willingness to continue treatment within this framework. If the patient was discharged from the unit because of an improved condition or due to transition to long term care, the unit staff provided information on the patient to the treating physician by direct personal communication in addition to a discharge letter.

### Data collection

Data, collected retrospectively, included data extraction from the patient’s computerized medical record for patients who joined home care from January 1, 2012 to December 31, 2014.

It included socio-demographic information such as age and sex, co-morbidity and burden of co-morbid diseases by the Charlson Comorbidity Index[24]. Data on the number of hospitalizations, the number of in-hospital days, emergency department visits, overall cost of healthcare, and mortality were obtained from the databases of Clalit Healthcare Services. A manual classification of the hospitalization was carried out by two of the authors (BP and RK) to decide if the hospitalization was related to CHF. The hospitalization was classified as CHF-related only if each author reached that conclusion independently.

The overall cost of healthcare included the cost of hospitalization, emergency room visits, laboratory tests, imaging procedures, consultations and the cost of the unit staff’s work. There was no patient co-payment for treatment in the unit.

### Data analyses

The monthly rate of emergency department visits, hospitalizations, number of in-hospital days related to CHF, and overall cost for the last six months before the patient entered the home care program were compared to the monthly rate of these variables after entry into the program. Use of the six-month period prior to entry into home care was based on the assumption that in this period the patient’s condition had deteriorated leading to admission to home care. In addition, a comparison was conducted on these variables between patients who died while in home care with those who survived.

Categorical variables, such as sex, country of origin, and chronic comorbidity are described as frequencies and percentages. Continuous variables such as age are described as means and standard deviations. The comparison of healthcare utilization before and during home care was conducted by the Wilcoxon test for non-parametric repeated measurements, and presented as the median (interquartile range). The comparison between patients who died and those who survived was performed by the chi-square tests for categorical variables and t-tests for continuous variables with normal distribution (for example, age) or the Mann-Whitney Test for continuous variables with skewed distribution (for example, number of hospitalizations).

The study was approved by the Helsinki Committee of the Meir Medical Center (approval 0073-15com2). It was exempted by the committee from the need to have patients sign informed consent.

## Results

Over the study period, from 2012–2014, 196 CHF patients were treated in the unit. Of these, 113 (57.7%) were women. The mean age was 79.4±9.5 and the mean length of home care was 614.1±368.2 days. Eighty patients (40.8%) were classified as NYHA class 4, and 116 (59.2%) as class 3. One hundred and five patients died over the study period (53.6%). Patient characteristics are presented in Table 1.

**Table 1:** Characteristics of the study population (N = 196).

		N	%
**Sex**	Male	83	42.3%
	Female	113	57.7%
**Age**	49–65	5	2.6%
	65+	191	97.4%
	Mean ± SD	79.4±9.5	
	Range	49–103.7	
**Days in home care**	Mean ± SD	614.07±368.2	
	Range	83–1705	
**Died during home care period**	Yes	105	53.6%
	No	91	46.4%
**CCITS**	Mean ± SD	3.8±1.8	
**Co-morbidity**	S/p MI	80	40.8%
	S/p CVA	60	30.6%
	Dementia	36	18.4%
	COPD	40	20.4%
	DM	104	53.1%
	CRF moderate to severe	22	11.2%
	Solid Tumor	17	8.7%
	HTN	175	89.3%
**NYHA**	NYHA class 3	116	59.2%
	NYHA class 4	80	40.8%
**Geriatric syndromes**	Incontinence	38	19.4%
	Falls	41	20.9%
	Bed ridden	24	12.2%
	Pressure sore	14	7.1%

**CCITS**- Charlson Comorbidity Index Total Score, **MI**-myocardial infarction, **CVA**-cerebro-vascular accident, **COPD** -chronic obstructive pulmonary disease, **DM**-diabetes mellitus, **CRF**-chronic renal failure, **HTN**-hypertension, **NYHA**- New York Heart Association

### Utilization of healthcare services

There was a statistically significant drop in the monthly number of hospitalizations, the number of total hospital days for CHF, the number of emergency department visits, and the trend in overall cost reduction in comparison with the corresponding monthly rates for the six months prior to entrance into the framework of home care (Table 2).

**Table 2:** Comparison of mean healthcare utilization and costs, per patient, before and after entry into home care.

	Half year prior to entry	During home care	Change (%)	P-value
Number of monthly hospitalizations Median (IQR)	0.333 (0.167–0.500)	0.147 (0.049–0.244)	55.8%	<0.0001
Number of monthly hospital days Median (IQR)	1.417 (0.333–3.667)	0.831 (0.244–1.797)	41.35%	<0.0001
Number of monthly hospital days for CHF Median (IQR)	0.833 (0–2.134)	0.516 (0–0.636)	-79.7%	<0.0001
Number of monthly emergency room visits Median (IQR)	0 (0–0.167)	0 (0–0.049)		<0.0001
Monthly cost (US$) Median (IQR)	870.15 (247.2–1879.1)	690 (255.3–1346.5)	-20.7%	0.056

**IQR**-interquartile range

There were no significant differences between patients who died and those who survived in terms of age, sex, burden of comorbidity (CCI), rate of specific diseases (except for moderate to severe renal failure), rate of geriatric syndromes, percentage of patients classified as NYHA 3 or 4, number of monthly unit team visits (except doctor visits), and mean total number of visits (Table 3).

**Table 3:** Comparison between patients who survived and patients who died.

		Survived (N = 91)		Died (N = 105)		P-value
		N	%	N	%	
**Sex**	Male	34	37.4%	49	46.7%	0.20
	Female	57	62.6%	56	53.3%	
**Age**	SD±Mean	80.0±8.8		78.9±10.3		0.72
	Range	49–103.7		59–98		
**Duration of home care (days)**	SD±Mean	826.7±341.4		428.9±286.3		<0.002
	Range	110–1704		82–1288		
**CCITS**	SD±Mean	3.8±1.8		3.9±1.8		0.49
	Range	1–8		1–9		
**Comorbidity**	S/p MI	34	37.4%	46	43.8%	0.385
	S/p CVA	32	35.2%	28	26.7%	0.216
	Dementia	15	16.5%	21	20.0%	0.582
	COPD	18	19.8%	22	21.0%	0.861
	DM	45	49.5%	59	56.2%	0.588
	CRF moderate to severe	5	5.5%	17	16.2%	0.01
	Solid Tumor	9	9.9%	8	7.6%	0.701
	HTN	78	85.7%	97	92.4%	0.166
	Falls	20	22.0%	21	20.0%	0.86
	Bed ridden	10	11.0%	14	13.3%	0.669
	Incontinence	16	17.6%	22	21.0%	0.591
	Pressure Ulcers	6	6.6%	8	7.6%	0.999
**NYHA class**	Class 3	52	57.1%	64	60.9%	0.677
	Class 4	39	42.9%	41	39.1%	
**Mean number of monthly visits in home care**	Doctors	3.3±1.0		3.6±1.4		0.016
	Nurses	5.2±3.9		5.8±3.9		0.222
	All others	0.3±0.4		0.4±0.7		0.217
	Total number of visits	8.8±4.5		9.8±4.6		0.056
**Mean number of monthly hospitalizations per patient**	Before home care	0.26±0.33		0.48±0.36		<0.0001
	During home care	0.21±0.27		0.2±0.19		0.463
	Difference (%)	-19.2%		-59.2%		
**Mean monthly number of hospital days per patient**	Before home care	1.4±2.1		3.2±3.0		<0.0001
	During home care	1.02±1.7		1.74±1.9		0.083
	Difference (%)	-29.20%		-44.90%		
**Mean monthly overall cost per patient ($)**	Before home care	776.9±1301		1639±1469		<0.0001
	During home care	497.9±1129		1328±1134		<0.0001

**CCITS**- Charlson Comorbidity Index Total Score, **MI**-myocardial infarction, **CVA**-cerebro-vascular accident, **COPD** -chronic obstructive pulmonary disease, **DM**-diabetes mellitus, **CRF**-chronic renal failure, **HTN**-hypertension,

The mean length of stay in home care for patients who died was 428.9±286.3 compared to 826±341.4 for those who survived (p<0.002).

The mean number of physician visits to the home care patient was 3.5±1.2 per month. The corresponding number for nurses was 5.5±3.9 and for other unit providers (nutritionist, social worker, physical and occupational therapists) 0.3±0.6. The mean monthly number of hospitalizations, the mean monthly number of hospital days and the mean overall costs before entry into home care were higher among the patients who died compared to those who survived. A statistically significant decrease of 59.2% was found in the mean monthly number of hospitalizations per patient in the patients who died (0.48±0.36 vs. 0.2±0.19, p<0.0001), but not in patients who survived (0.26±0.33 vs. 0.21±0.27, p = 0.207). It is noteworthy that among two groups of the patients over the course of home care there was a statistically significant decline in the number of hospitalization days per month per patient (1.4±2.1 vs 1.02±1.7, p<0.0001 in the patients who survived and 3.2±3.0 vs. 1.74±1.9, p<0.0001 in the patients who died). Furthermore, there was a decrease in overall cost after entering the home care program in both groups (776.9$±1301$ vs. 497.9$±1129$, p<0.0001 among the patients who survived and 1639$±1469$ vs 1328$±1134$, p<0.0001among the patients who died) (Table 3).

## Discussion

The results of the present study show that among homebound CHF patients a treatment protocol implemented by a multidisciplinary team in the framework of home care reduces the rate of hospitalization and the number of hospital days, and the rate of emergency room visits without additional overall costs after adjustment for the cost of the unit team’s home visits. This reduction in the hospitalization rate and the rate of emergency department visits is consistent with the results of previous studies[10, 15, 19–21, 25–28]. In terms of cost effectiveness, the results of the present study are similar to other studies that also demonstrated economic benefits from similar interventions[28–30]. As in previous studies[28, 31, 32] the study population consisted of CHF patients 65 years of age and above with NYHA Class 3–4 severity and repeat, prolonged hospitalizations.

The present intervention program was different from most of the others[10, 15, 17, 20, 21, 25–33] in several parameters. First, all study participants were homebound and incapable of accessing various medical settings for their required treatment. Second, the treatment team included, in all cases, a doctor and a nurse who conducted home visits at least weekly. Third, in contrast to most of the other intervention programs[10, 15, 20, 21, 29] most of the patients were assessed by a geriatrician over the course of therapy.

We compared patients who died during the course of the study with those who survived. To our surprise, except for the finding that the patients who died had a higher rate of moderate to severe chronic renal failure (creatinine ≥ 3.0 mg/dl) there were no differences between these two groups in terms of age, sex, CCI, or NYHA classification. In any event, the patients who died were more sick as reflected in the higher rate of hospitalization prior to and during the intervention program. This raises questions as to the prognostic relevance of CCI in this complex population.

Before home care, the mean monthly number of hospital days per patient in the group of patients who died was significantly higher than in the survivor group (1.4±2.1vs.3.2±3.0, p<0.0001). However, the reduction in the number of hospital days in the deceased patients group was 44.9% compared to 29.2% in the group of patients who survived. Compared to the period before home care there was statistically significant decrease of 59.2% in the mean number of hospitalizations in the group of patients who died in the period after entry into home care, while the corresponding decrease in the group of survivors was not statistically significant (Table 3).

This outcome may indicate that home care may be particularly effective in the group of patients with more significant morbidity and a higher hospitalization rate. This finding could be significant for the development of home care programs in settings where the economic and human resources are limited and the policy decision-makers have to choose a specific target population. In those cases, admitting patients with a history of long hospitalizations prior to home care might be a beneficial policy in economic terms.

The advantages of the present study include its comprehensive database of socio-demographic data, chronic comorbidity and burden of illness (CCI), detailed information on hospitalization and emergency department visits, information on geriatric syndromes, and detailed data on the overall cost of treatment.

The study also has limitations. First, since this was a retrospective study there was no control group. The study group was comprised of those patients who were recruited into the intervention program because they were homebound, had significant CHF and needed intravenous therapy, which could not be administered in the community clinic. There was no ethical way to conduct a prospective study with a control group because there was no feasible alternative to home care to achieve clinical improvement. To overcome this limitation and to assess the effectiveness of the intervention program we compared healthcare utilization and overall cost between the 6 months prior to entrance into home care and over the course of the intervention program. A 6-month period was adopted because during this period the patient’s condition deteriorated to the point where they needed home care. However, it did not enable us to control for confounders, as would have been the case with a control group.

Second, the method of calculation that was used to compare the half-year prior to entry into home care with the period after entry was not ideal. For example, a hospitalization that occurred before entry into home care could have changed the balance towards proving the effectiveness of the program. In an attempt to control for the effect of the timing of the last hospitalization we conducted further sub-analyses in which we compared two sub-groups of study participants. The first group was patients whose entry into home care was less than 30 days after their last hospitalization (78 patients) and the second group was patients whose entry into home care was at least 30 days after their last hospitalization (92 patients). In both groups we found a decrease in the number of hospitalizations per month and in the monthly cost between the period before and after entry into the home care program. The rate of decrease was higher in the first group. Furthermore, 26 of the 196 patients who participated in the study did not have any hospitalization in the six months prior to their entry into home care, which may have attenuated the effect of the program.

Third, some home care teams did not record the patients’ functional state, despite repeated requests, so we could not analyze this important variable. On the other hand, the high rate of geriatric syndromes and the fact that almost 10% of the patients were bed-ridden provides indirect evidence for the impaired functional state of the study population. Another limitation of the present study, which also stems from its retrospective nature, is the lack of data on the effect of the program on the quality of life of the patients and their principal caregivers. The relatively small sample size is additional problem.

In summary, the results of this study support the conclusion that a home care intervention program, implemented by a multidisciplinary team, can reduce healthcare utilization and costs. It is reasonable to assume that this type of program would be even more effective among patients with higher rates of healthcare service utilization.

## Supporting information

S1 Table Data. Study Data.
(XLSX)
